# Primary Care Health Professional Shortage Area Designations Before and After the Affordable Care Act’s Shortage Designation Modernization Project

**DOI:** 10.1001/jamanetworkopen.2021.18836

**Published:** 2021-07-30

**Authors:** Christopher A. Scannell, Jacob K. Quinton, Nicholas J. Jackson, Yusuke Tsugawa

**Affiliations:** 1 VA Greater Los Angeles Healthcare System, Los Angeles, California; 2 National Clinician Scholars Program, University of California, Los Angeles (UCLA); 3 Division of General Internal Medicine and Health Services Research, David Geffen School of Medicine at UCLA, Los Angeles; 4 Department of Health Policy and Management, UCLA Fielding School of Public Health, Los Angeles

## Abstract

This cross-sectional study evaluates whether the Patient Protection and Affordable Care Act (ACA) Shortage Designation Modernization Project is associated with changes in primary care health professional shortage area (PC-HPSA) designations.

## Introduction

Primary care physician (PCP) supply in the United States is unequal and diverging, with PCP density in urban counties double that of rural counties, and more than 50% of rural counties losing PCPs in the past decade.^1^ To help reverse this pattern, the federal government provides incentives, such as loan repayment programs, bonus payment programs, or programs to recruit noncitizen foreign medical graduates, for physicians practicing in primary care health professional shortage areas (PC-HPSAs), which are federally designated areas with inadequate PCP access.^2^ A county may be designated a partial-county PC-HPSA if populations or facilities with high needs exist at a subcounty level or a full-county PC-HPSA if the entire county meets a minimum population-to-PCP ratio of 3000 to 1 with high need or a ratio of 3500 to 1 in the absence of high need.^3^ These designations are time intensive to document and thus are updated infrequently, leading to inaccurate designations and misaligned incentives for physicians.^4^ The Shortage Designation Modernization Project (SDMP) was implemented in 2014 to streamline designations as part of the Patient Protection and Affordable Care Act.^5^ Whether SDMP implementation is associated with changes in PC-HPSA designations and whether these changes accurately reflect county-level physician supply are unknown.

## Methods

In this cross-sectional study, we used the Health Resources and Services Administration Area Health Resources Files and extracted county-level PC-HPSA designations, population, and PCP counts (originally derived from the American Medical Association Physician Masterfile) from 2010 to 2018. Data were analyzed from November 2020 to April 2021. The University of California, Los Angeles Institutional Review Board deemed this study exempt from review and waived the requirement for informed consent based on the use of publicly available, deidentified data. The Strengthening the Reporting of Observational Studies in Epidemiology (STROBE) reporting guideline was followed.

Temporal trends in PC-HPSA designations and population-to-PCP ratios were examined. Segmented regression analysis for 2 periods, before SDMP implementation (2010-2013) and after SDMP implementation (2015-2017), excluding the implementation year (2014), was used to examine the association between SDMP implementation and population-to-PCP ratio. Covariates included year and county PC-HPSA designation and their interaction with the policy change. Projected population-to-PCP ratios in 2015 were compared in the presence and absence of the SDMP. All statistical tests were 2-sided, with a significance threshold of *P* < .05 and were conducted using Stata/SE, version 16.0 (StataCorp, LLC).

## Results

**Figure 1.**
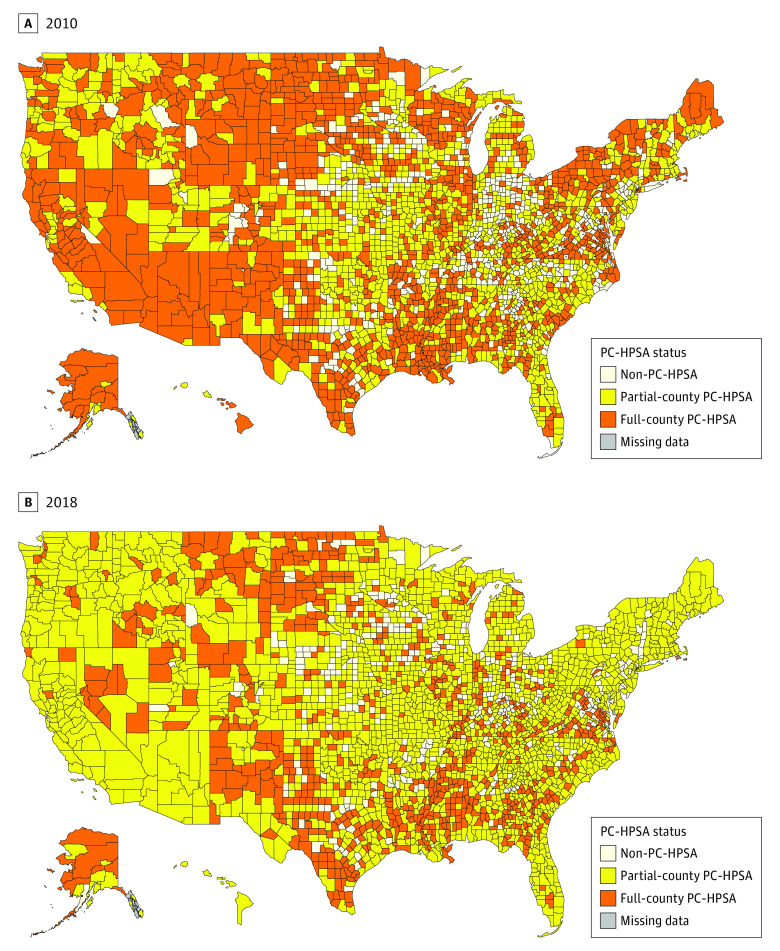
Geographic Distribution of Primary Care Health Professional Shortage Area (PC-HPSA) Designations County-level distribution PC-HPSA designations in 2010 (A) and 2018 (B). The maps are color-coded based on designation status or where data are missing.

**Figure 2.**
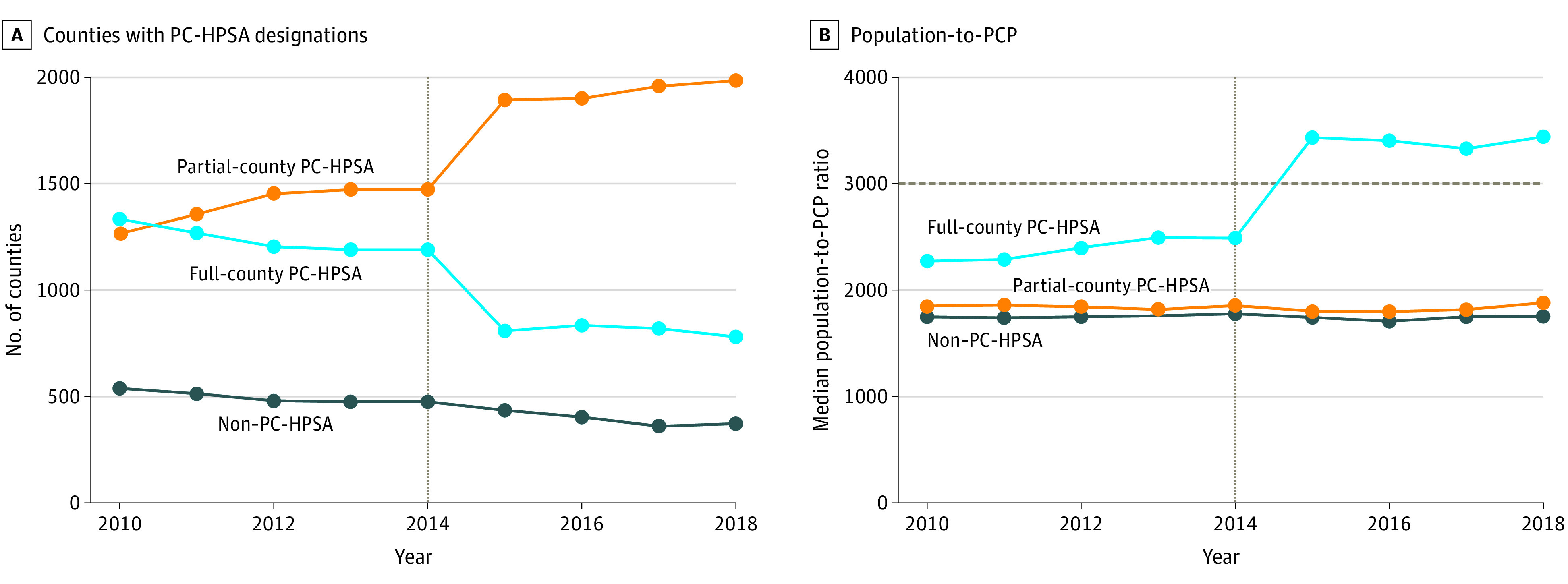
Temporal Changes in Primary Care Health Professional Shortage Area (PC-HPSA) Designations and Population-to-Primary Care Physician (PCP) Ratios Line graphs of the annual number of counties with non-, partial-county, and full-county PC-HPSA designations (A) and the corresponding median population-to-PCP ratios within these counties between 2010 and 2018 (B). The gray vertical line denotes 2014, when the Shortage Designation Modernization Project was implemented. In panel B, the dashed line indicates the minimum required 3000 to 1 population-to-PCP ratio for full-county PC-HPSA designation.

There were 3137 counties with complete data across the observational period (538 non-PC-HPSAs [17%], 1334 partial-county PC-HPSAs [42%], and 1265 PC-HPSAs [40%] beginning in 2010). County-level designations changed between 2010 and 2018, with many full counties redesignated as partial-county PC-HPSAs after SDMP implementation (Figure 1). The number of non–PC-HPSA and full-county designations immediately before (2013) and after (2015) SDMP implementation decreased by 8% and 32%, respectively. The number of partial-county PC-HPSAs increased by 29% (Figure 2A), with an increase in the median population-to-PCP ratio for full-county PC-HPSAs greater than the minimum required ratio of 3000 to 1. The population-to-PCP ratio for partial-county or non-PC-HPSAs remained constant (Figure 2B). Segmented regression estimates aligned with the descriptive findings: a significant increase was found in the population-to-PCP ratio in full-county PC-HPSAs after implementation of the SDMP compared with before implementation (difference: 293 person-to-PCP increase [95% CI, +176 to +410]; *P* < .001). No other significant findings were found.

## Discussion

The Patient Protection and Affordable Care Act has changed the US health care delivery system in many ways that have yet to be evaluated, including streamlining the designation of PC-HPSAs, which may more appropriately incentivize PCPs to practice in underresourced settings. The Shortage Designation Modernization Project implementation coincided with a change in PC-HPSA designations from full-county to partial-county PC-HPSAs, sharpening the geographic focus of the designations to accurately reflect areas with low PCP supply. Additional research is warranted to understand the implications of the SDMP for the distribution of incentive payments, and subsequently PCPs, and to ascertain whether this policy change has effectively redirected resources to underresourced areas. In the future, incorporation of additional deprivation indicators into the PC-HPSA designation process may be needed to better incentivize health care access for patients in areas with high social and medical needs.

This study has limitations, including the use of county-level data, which do not address changes in other types of PC-HPSA designations based on populations or facilities at the subcounty level. We also used physician counts derived from the American Medical Association Physician Masterfile to calculate population-to-PCP ratios, which may overestimate the current number of practicing physicians.^6^ However, this overestimation would produce an underestimation of the population-to-PCP ratios, suggesting even greater physician shortages in the full-county PC-HPSAs identified after SDMP implementation.

## References

[1] Machado SR, Jayawardana S, Mossialos E, Vaduganathan M. Physician density by specialty type in urban and rural counties in the US, 2010 to 2017. JAMA Netw Open. 2021;4(1):e2033994. doi:10.1001/jamanetworkopen.2020.33994 33481030 PMC7823223

[2] Health Resources & Services Administration Health Workforce. What is shortage designation? Accessed May 13, 2021. https://bhw.hrsa.gov/workforce-shortage-areas/shortage-designation

[3] Electronic Code of Federal Regulations. Part 5—designation of health professional(s) shortage areas. Accessed November 13, 2020. https://www.ecfr.gov/cgi-bin/retrieveECFR?gp=&SID=994659af422559ab44267d5729954544&mc=true&n=pt42.1.5&r=PPAR&ty=HTML#ap42.1.5_14.a

[4] US Government Accountability Office. Health care shortage areas: designations not a useful tool for directing resources to the underserved. September 1995. Accessed May 13, 2021. https://www.govinfo.gov/content/pkg/GAOREPORTS-HEHS-95-200/html/GAOREPORTS-HEHS-95-200.htm

[5] Health Resources & Services Administration Health Workforce. Understanding the Shortage Designation Modernization Project. Accessed November 2, 2020. https://bhw.hrsa.gov/shortage-designation/application-review-process/modernization-project

[6] Staiger DO, Auerbach DI, Buerhaus PI. Comparison of physician workforce estimates and supply projections. JAMA. 2009;302(15):1674-1680. doi:10.1001/jama.2009.1461 19843902 PMC2791886

